# Validity of the Stages of Change in Steps instrument (SoC-Step) for achieving the physical activity goal of 10,000 steps per day

**DOI:** 10.1186/s12889-015-2539-y

**Published:** 2015-11-30

**Authors:** Richard R. Rosenkranz, Mitch J. Duncan, Cristina M. Caperchione, Gregory S. Kolt, Corneel Vandelanotte, Anthony J. Maeder, Trevor N. Savage, W. Kerry Mummery

**Affiliations:** Kansas State University, Manhattan, USA; Western Sydney University, Sydney, Australia; University of Newcastle, Newcastle, Australia; University of British Columbia, Kelowna, Canada; Central Queensland University, Rockhampton, Australia; University of Alberta, Edmonton, AB Canada

**Keywords:** Motivation, Transtheoretical Model, Targeted intervention, Sedentary lifestyle, Walking, Pedometer

## Abstract

**Background:**

Physical activity (PA) offers numerous benefits to health and well-being, but most adults are not sufficiently physically active to afford such benefits. The 10,000 steps campaign has been a popular and effective approach to promote PA. The Transtheoretical Model posits that individuals have varying levels of readiness for health behavior change, known as Stages of Change (Precontemplation, Contemplation, Preparation, Action, and Maintenance). Few validated assessment instruments are available for determining Stages of Change in relation to the PA goal of 10,000 steps per day. The purpose of this study was to assess the criterion-related validity of the SoC-Step, a brief 10,000 steps per day Stages of Change instrument.

**Methods:**

Participants were 504 Australian adults (176 males, 328 females, mean age = 50.8 ± 13.0 years) from the baseline sample of the Walk 2.0 randomized controlled trial. Measures included 7-day accelerometry (Actigraph GT3X), height, weight, and self-reported intention, self-efficacy, and SoC-Step: Stages of Change relative to achieving 10,000 steps per day. Kruskal-Wallis H tests with pairwise comparisons were used to determine whether participants differed by stage, according to steps per day, general health, body mass index, intention, and self-efficacy to achieve 10,000 steps per day. Binary logistic regression was used to test the hypothesis that participants in Maintenance or Action stages would have greater likelihood of meeting the 10,000 steps goal, in comparison to participants in the other three stages.

**Results:**

Consistent with study hypotheses, participants in Precontemplation had significantly lower intention scores than those in Contemplation (*p* = 0.003) or Preparation (*p* < 0.001). Participants in Action or Maintenance stages were more likely to achieve ≥10,000 steps per day (OR = 3.11; 95 % CI = 1.66,5.83) compared to those in Precontemplation, Contemplation, or Preparation. Intention (*p* < 0.001) and self-efficacy (*p* < 0.001) to achieve 10,000 steps daily differed by stage, and participants in the Maintenance stage had higher general health status and lower body mass index than those in Precontemplation, Contemplation and Preparation stages (*p* < 0.05).

**Conclusions:**

This brief SoC-Step instrument appears to have good criterion-related validity for determining Stages of Change related to the public health goal of 10,000 steps per day.

**Trial registration:**

Australian New Zealand Clinical Trials Registry reference: ACTRN12611000157976 World Health Organization Universal Trial Number: U111-1119-1755

**Electronic supplementary material:**

The online version of this article (doi:10.1186/s12889-015-2539-y) contains supplementary material, which is available to authorized users.

## Background

Maintaining a physically active lifestyle offers numerous benefits to health and well-being [1]. A large proportion of adults, however, are not sufficiently physically active to afford such benefits [2]. Although physical activity guidelines are typically presented in terms of minutes per week [1], communicating physical activity in terms of steps per day has been offered as an easily understood standard that can be assessed with pedometers or other inexpensive physical activity monitors [3]. Pedometers can be useful tools for motivation, goal setting, and self-monitoring; a systematic review of physical activity promotion studies employing pedometers showed that their use, within a broader behavior change intervention, was associated with increased physical activity, as well as improvements in blood pressure and body mass index [4].

Many public health interventions have employed pedometers or other step-counters while targeting the promotion of adults’ physical activity levels, and 10,000 steps per day campaigns have been popular and effective approaches in these efforts [5–9]. Although the 10,000 steps per day goal is not universally appropriate across various levels of age, gender, and physical function, the goal is deemed to be a reasonable and motivating target for healthy adults [3]. Physical activity intervention studies employing the 10,000 steps per day goal have shown weight loss, improved glucose tolerance, and reduced blood pressure among the outcomes from increased physical activity toward achieving this goal [10, 11].

Researchers and public health practitioners have recognized that participants in health promotion programs have varying levels of readiness to undertake potentially challenging lifestyle changes, such as increasing physical activity to a level of 10,000 steps per day [12–16]. Consistent with the Transtheoretical Model [12], interventions can target various subgroups, based on level of readiness for health behavior change, and interventions can be tailored to differing needs or preferences of the participants [13–17]. Studies have shown that such targeted approaches are effective [13], cost-effective [14, 15] and often lead to greater improvements in health outcomes compared to non-targeted or non-tailored approaches [13, 16].

Tailored interventions are increasingly common, and have shown success in increasing physical activity [13–17]. Few validated assessment instruments are currently available for determining Stages of Change according to the Transtheoretical Model, and none has been based around the 10,000 steps per day public health physical activity goal. The purpose of this study was to assess the criterion-related validity of a brief 10,000 steps per day Stages of Change in Steps (SoC-Step) instrument. To achieve this, we used baseline data from the Walk 2.0 Study, a 3-arm randomized controlled trial addressing the effects of Web 2.0 applications on engagement, retention, and physical activity behavior change [18].

### Hypotheses

According to the Transtheoretical Model, individuals can be categorized into one of five stages, based on the combination of behavioral intention and previous behavior. Therefore, our main hypothesis testing focused on each of these aspects from the SoC-Step instrument: whether or not participants indicated that they intended to be, and/or indicated currently being, active at a level of taking 10,000 steps per day. Consistent with previous literature [12, 19, 20], it was hypothesized that participants who self-categorized as being in Precontemplation on the SoC-Step instrument would have significantly lower intention scores compared to participants who self-categorized into Contemplation or Preparation stages. Regarding previous behavior, it was hypothesized that those self-categorized into Action and Maintenance stages would have greater levels of accelerometer-measured steps per day, compared to those in Precontemplation, Contemplation, or Preparation stages. In a similar vein, it was hypothesized that if the SoC-Step instrument had good criterion validity, participants in the Action and Maintenance stages, as compared to those in Precontemplation, Contemplation, or Preparation stages, would have significantly higher likelihood to reach the 10,000 steps per day goal.

In addition to the core elements of intention and previous behavior, the Transtheoretical Model suggests that self-efficacy increases as individuals move from Contemplation to Preparation and Action stages. Therefore, we hypothesized that self-efficacy to be physically active at a level of taking 10,000 steps per day would differ among these three stages, with Action showing the highest levels of self-efficacy. Also, given that there is an abundance of literature on the health benefits of regular physical activity [1], we hypothesized that participants in the Maintenance stage would have significantly higher levels of general health and lower body mass index values, as compared to those in Precontemplation, Contemplation, and Preparation.

## Methods

### Participants

Participants were 504 Australian adults (176 males, 328 females, mean age = 50.8 ± 13.0 years) from the baseline sample of the Walk 2.0 randomized controlled trial [18]. This three-arm randomized controlled trial aimed to evaluate the effectiveness of two web-based physical activity interventions, compared to a control group (using a physical activity log book). Both interventions encouraged meeting the 10,000 steps per day goal (or an equivalent level of any physical activity), but also allowed for individual goals above or below that standard. Full details of recruitment methods and the study protocol are published elsewhere [18]. The study received ethics approval from the Human Research Ethics Committees of the University of Western Sydney (Reference number H8767) and CQUniversity (H11/01-005).

To be eligible, participants were residents of Western Sydney (New South Wales) or Rockhampton (Queensland), Australia and were able to speak and read English. Eligibility requirements also included: being free of a medical condition which could be exacerbated by physical activity; being over 18 years of age; having access to the Internet but not a member of the website www.10000steps.org.au; and not currently meeting physical activity guidelines, but willing to increase activity levels.

### Psychosocial measurement

The current study draws upon questionnaire data that were obtained from an online survey that participants completed during baseline assessments. The online questionnaire included items and scales from the Transtheoretical Model [12, 20], Social Cognitive Theory [21, 22], and Theory of Planned Behavior [23], all relevant to the 10,000 steps per day goal. From the Transtheoretical Model, Stages of Change were determined using the SoC-Step instrument by asking whether participants were currently taking 10,000 steps per day, how long they had been doing so, or if they were not yet doing so, whether they intended to, or were preparing or beginning to take 10,000 steps per day. The SoC-Step instrument (see Additional file 1) was modelled on a previously published scale [20], but adapted to the 10,000 steps goal for the Walk 2.0 project.

Relevant to Social Cognitive Theory, the online questionnaire included 4 items (on a 0–100 scale; Cronbach’s α = 0.848) to assess self-efficacy for physical activity [22] and 10 items (on a 0–100 scale; Cronbach’s α = 0.923) to assess self-efficacy to overcome common barriers to physical activity [22]. Self-efficacy items required participants to rate their degree of confidence for a set of incremental physical activity behavioral targets (2,000 steps per day; 6,000 steps per day; 10,000 steps per day; 14,000 steps per day) by recording a number from 0 (cannot do at all) to 100 (highly certain can do). Relevant to the Theory of Planned Behavior, the online questionnaire included 2 items (on a 1–5 scale; Cronbach’s α = 0.832) on intention and 4 items (on a 1–5 scale; Cronbach’s α = 0.772) on attitudes related to being physically active at a level of taking 10,000 steps per day. These were based on a set of previously published items [24], but were modified for Walk 2.0 project needs toward the behavioral goal of 10,000 steps. Intention items rated on a 5-point scale were, “I intend to be physically active at a level of taking 10,000 steps on most days, if not all days of the week, for the next month” and “I will try to be physically active at a level of taking 10,000 steps on most days, if not all days of the week, for the next month.”

Other online questionnaire scales included the RAND 36 item Short Form Health Survey (SF-36) to assess quality of life [25]. This instrument covers 8 dimensions of health, including limitations in physical activities and usual role activities due to health problems, bodily pain, general mental health, and vitality (energy and fatigue). The SF-36 has demonstrated adequate validity in Australian populations [26], and is suitable for use in the general population of adults [27, 28].

### Physical activity monitoring

Physical activity was assessed with ActiGraph GT3X physical activity monitors (ActiGraph, Shalimar, FL, USA). ActiGraph monitors have demonstrated reliability and validity for the measurement of physical activity, including step counts, in free-living environments [29–31]. Participants were provided with an ActiGraph (fastened to an elastic belt) which they were asked to wear on their right hip. Participants were instructed to wear the ActiGraph continuously for 7 full days during waking hours, unless they planned to swim, bathe, or play contact sports. Participants were asked to complete a physical activity log that was used to record activities undertaken when the ActiGraph was not worn and the duration of non-wear.

ActiGraph data were collected in 1-s epochs, including step counts. When participants returned to complete baseline assessments (at least 8 days after receiving the ActiGraph) the physical data were downloaded and stored. Wear time was assessed using the criteria of 60 min of consecutive zero data with a 2-min spike tolerance [32]. Minimum valid wear time was set at 600 min of wear time per day across a minimum of 5 days. Participants with insufficient valid wear time were asked to wear the ActiGraph for an additional 7 days. Those not returning an ActiGraph that contained at least minimal valid wear time data in three attempts (*n* = 39) were excluded from analysis. Mean wear time among participants was 867.2 (SD = 73.7) minutes per day, recorded across 5.9 (SD = 0.7) valid days. Outcome variables for the present study consisted of steps per day (adjusted for total accelerometer wear time), and whether or not the daily step count met the 10,000 steps per day goal.

### Anthropometry

Height, weight, and waist circumference were measured by project staff in a research setting at baseline assessments. Weight was measured in light clothing and without shoes using Seca 700 mechanical scales (Seca Corp., Hamburg, Germany). Height was measured with feet together and head held in the Frankfurt plane, via Seca 220 measuring rod (Seca Corp., Hamburg, Germany). Waist circumference was measured using Seca 203 measurement tape (Seca Corp., Hamburg, Germany) at the plane aligned with both iliac crests, in accordance with the National Institutes of Health protocol [33].

### Statistical analysis

All analyses were conducted using SPSS for Windows (Version 22.0), with alpha set at <0.05. Variables were checked for normality and other parametric assumptions, and non-parametric tests were used when assumptions were violated. Descriptive statistics included frequencies, percentages, mean, median, standard deviation, and inter-quartile range.

Independent samples Mann–Whitney or Kruskal Wallis tests (including pairwise comparisons where necessary) were used to test:

1. The hypothesis that participants in Precontemplation would have significantly lower intention scores compared to participants in Contemplation or Preparation stages;
2. The hypothesis that those in Action or Maintenance stages would have greater levels of accelerometer-measured physical activity compared to those in Precontemplation, Contemplation, or Preparation stages;
3. The hypothesis that self-efficacy would differ among Contemplation, Preparation, and Action stages, with Action showing the highest levels of self-efficacy;
4. The hypothesis that participants in the Maintenance stage would have significantly higher levels of general health and lower body mass index values, as compared to those in Precontemplation, Contemplation, and Preparation stages.

Binary logistic regression, with odds ratios and 95 % confidence intervals, was used to test the hypothesis that participants in Maintenance or Action stages would have greater likelihood of meeting the 10,000 steps goal, in comparison to participants in the other three stages.

## Results

Demographics for the sample are shown in Table 1. Participants comprised mostly middle-aged adults (mean BMI over 29 kg/m^2^; mean age around 51 years old); the majority of participants were women (65 %) and were overweight or obese (75 %). More participants were recruited from the Rockhampton area (*n* = 311) than from major metropolitan area of Western Sydney (*n* = 193). The sample was diverse with regard to education, income, employment, and physical activity level, but the vast majority was born in Australia (79 %) and spoke English at home (85 %).

**Table 1:** Descriptive characteristics of Walk 2.0 study participants

	*N*	Mean ± SD
Age (years)	504	50.8 ± 13.1
Height (m)	504	1.67 ± 0.09
Weight (kg)	504	81.8 ± 18.9
Body mass index (kg/m2)	504	29.3 ± 6.0
Waist circumference (cm)	504	99.9 ± 15.0
Physical activity level (unadjusted steps/day)	465	7,248 ± 2,424
	*N*	Percentage
Sex		
Males	176	34.9 %
Females	328	65.1 %
Weight Status		
Underweight	6	1.2 %
Normal weight	117	23.2 %
Overweight	181	35.9 %
Obese	200	39.7 %
Education		
Postgraduate degree	40	7.9 %
Graduate certificate or diploma	38	7.5 %
Bachelor degree	93	18.5 %
Advanced diploma/diploma	75	14.9 %
School certificate	118	23.4 %
School education	140	27.8 %
Household combined annual income (AUD)		
$0—$41,599	111	20.0 %
$41,600—$77,999	117	23.2 %
$78,000—$129,999	115	22.8 %
$130,000+	103	20.4 %
Did not report income	68	13.5 %
Area of residence		
Sydney area	193	38.3 %
Rockhampton area	311	61.7 %
Employment		
Full time employment	234	46.4 %
Employed part-time/casual	111	22.1 %
Retired/Pensioner	96	19.0 %
Other	63	12.5 %
Country of birth		
Australia	398	79.0 %
United Kingdom	35	6.9 %
India	8	1.6 %
Other	63	12.5 %
Speak language other than English at home	75	14.9 %

Table 2 displays participants’ health-related characteristics by Stages of Change. Descriptive statistics include means and standard deviations. Inferential statistics include the Kruskal-Wallis H test of distributions. These Kruskal-Wallis tests revealed that the distribution of all variables differed among the five Stages of Change (*p* < 0.05). The following are results of the specific tests of our hypotheses used to examine the validity of the SoC-Step instrument.

**Table 2:** Participants’ health-related variables by Stages of Change (*n* = 506)

	Precontemplation *n* = 16 Mean ± SD	Contemplation *n* = 201 Mean ± SD	Preparation *n* = 214 Mean ± SD	Action *n* = 13 Mean ± SD	Maintenance *n* = 60 Mean ± SD	*p*-value^a^
Steps per day^b^	7,147 ± 3,166	6,998 ± 2,119	7,077 ± 2,210	7,864 ± 2,550	8,464 ± 2,917	*p* = 0.015
Waist circumference	102.7 ± 13.5	100.6 ± 14.8	101.3 ± 15.2	100.5 ± 17.5	91.8 ± 12.8	*p < 0.001*
Body mass index	30.7 ± 5.5	29.7 ± 6.1	29.9 ± 6.0	28.3 ± 4.8	25.2 ± 4.1	*p < 0.001*
Intention	2.5 ± 1.3	3.8 ± 0.7	4.3 ± 0.6	4.0 ± 0.7	4.4 ± 0.6	*p < 0.001*
Self-efficacy PA	6.2 ± 2.8	8.1 ± 1.8	8.9 ± 1.6	9.9 ± 0.8	10.0 ± 1.1	*p < 0.001*
Self-efficacy barriers to PA	5.1 ± 2.4	6.5 ± 1.9	7.0 ± 1.9	7.5 ± 1.9	7.8 ± 1.6	*p < 0.001*
Attitudes	3.1 ± 1.0	4.0 ± 0.5	4.1 ± 0.5	4.1 ± 0.6	4.1 ± 0.7	*p < 0.001*
Physical functioning	76.3 ± 27.2	82.6 ± 18.2	85.9 ± 15.0	87.3 ± 17.5	94.8 ± 7.9	*p < 0.001*
Role physical	68.8 ± 38.2	78.7 ± 34.5	82.8 ± 30.5	76.9 ± 33.0	92.9 ± 16.7	*p* = 0.035
Energy fatigue	52.8 ± 17.9	53.2 ± 18.8	55.7 ± 20.5	66.2 ± 15.6	64.7 ± 18.7	*p* = 0.001
Emotional wellbeing	68.3 ± 19.8	75.4 ± 16.5	78.7 ± 14.8	78.8 ± 14.5	81.1 ± 13.8	*p* = 0.041
Role emotional	56.3 ± 48.3	78.3 ± 33.6	82.9 ± 30.6	87.2 ± 32.0	87.8 ± 28.1	*p* = 0.021
Social functioning	77.3 ± 25.1	85.1 ± 19.0	86.7 ± 19.0	81.7 ± 22.6	91.3 ± 17.0	*p* = 0.033
Pain score	72.7 ± 27.4	78.0 ± 20.9	77.7 ± 21.9	72.3 ± 29.8	87.6 ± 15.0	*p* = 0.015
General health	50.9 ± 16.1	62.0 ± 19.9	63.6 ± 20.0	68.8 ± 11.9	79.1 ± 15.0	*p < 0.001*

Note: Means and SD shown for descriptive purposes

^a^Exact *p* value is from Kruskal-Wallis test of overall difference among Stages of Change distributions (not based on means, due to data skewness)

^b^Steps per day adjusted for total accelerometer wear time

### Intention to be physically active at a level of 10,000 steps per day

Consistent with hypotheses, there were significant differences in intention scores between non-intenders (“I do NOT intend to be physically active at a level of taking 10,000 steps…” = Precontemplation stage) and those who intended (“I do intend to be physically active at a level of taking 10,000 steps…” = Contemplation and Preparation stages) to be more physically active (*H* = 70.9, *df* = 2, *p* < 0.001). Specifically, those participants who self-categorized into Precontemplation (median = 2.3, IQR = 1.1, 3.9), had significantly lower intention scores than those in Contemplation (median = 4.0, IQR = 3.5, 4.0; *H* = 3.3, *df* = 1, *p* = 0.003) or Preparation (median = 4.0, IQR = 4.0, 5.0; *H* = 5.9, *df* = 1, *p* < 0.001).

### Previous physical activity behavior: accelerometer-measured steps per day

Consistent with study hypotheses, results showed significant differences in accelerometer-measured steps per day between participants who self-categorized into Action or Maintenance stages (“Currently, I take enough steps [10,000 steps per day] to receive health benefits”), and those who self-categorized into Precontemplation, Contemplation, or Preparation (“Currently, I do NOT take enough steps [10,000 steps per day] to receive health benefits.”) stages (U = 17,273, *df* = 1, *p* < 0.001). Specifically, those participants who self-categorized into Action or Maintenance stages (median = 7,654, IQR = 6,386,10,172), had significantly higher step counts than those in Contemplation or Preparation (median for “intenders” = 6,724, IQR = 5,594, 8,295; *H* = 68.2, *df* = 1, *p* < 0.001) but not Precontemplation (median for “non-intenders” = 6,222; IQR = 4,863, 9,605; *H* = 83.1, *df* = 1, *p* = 0.124).

Also consistent with the study hypothesis, analysis revealed that those in Action or Maintenance stages were more likely to achieve 10,000 steps per day, as compared to those in Precontemplation, Contemplation, or Preparation stages (27 vs. 11 % of participants meeting the 10,000 steps goal; OR = 3.11; 95 % CI = 1.66, 5.83, *p* < 0.001).

### Physical activity self-efficacy

**Fig. 1:**
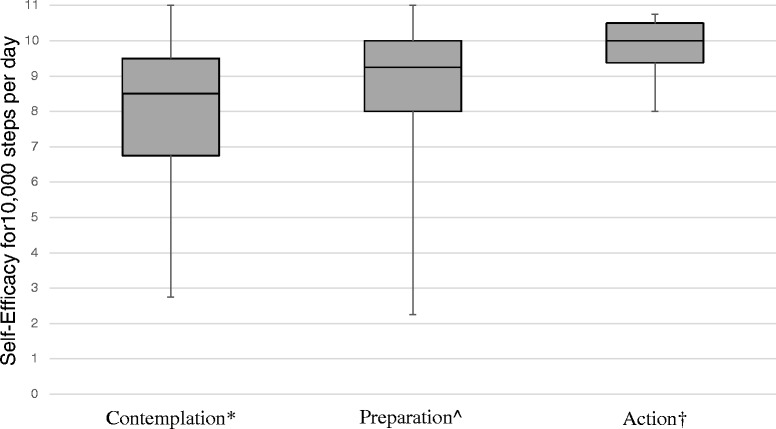
Self-Efficacy by Stages of Change. As hypothesized based on the Transtheoretical Model, self-efficacy levels differed across Contemplation, Preparation, and Action Stages (*H* = 34.8, *df* = 2, *p* < 0.001). * Contemplation differed significantly from Preparation (*H* = 2.4, *df* = 1, *p* < 0.001) and Action (*H* = 4.1, *df* = 1, *p* < 0.001) stages. ^ Preparation differed significantly from Contemplation (*H* = 4.9, *df* = 1, *p* < 0.001) and Action (*H* = 2.4, *df* = 1, *p* < 0.001) stages. † Action differed significantly from Contemplation (*H* = 4.1, *df* = 1, *p* < 0.001) and Preparation (*H* = 4.9, *df* = 1, *p* < 0.001) stages

Figure 1 shows self-efficacy to achieve 10,000 steps per day by Stages of Change. The stages differed in measures of self-efficacy to achieve 10,000 steps per day (*H* = 34.8, *df* = 2, *p < 0.001*). As hypothesized, pairwise comparisons showed that Action participants (median = 10.0; IQR = 9.4, 10.5) had significantly higher self-efficacy to achieve 10,000 steps per day than those in Contemplation (median = 8.5; IQR = 6.8, 9.5; *H* = 4.1, *df* = 1, *p* < 0.001), or Preparation (median = 9.3; IQR = 8.0, 10.0; *H* = 2.4, *df* = 1, *p* < 0.001). Preparation stage participants also had significantly greater self-efficacy than those in Contemplation (*H* = 4.9, *df* = 1, *p* < 0.001).

Similarly, stages differed in measures of self-efficacy to overcome common barriers to physical activity (*H* = 8.4, *df* = 2, *p* = 0.015). As hypothesized, pairwise comparisons showed that Preparation stage (median = 7.1; IQR = 5.7, 8.5) participants had higher self-efficacy than those in Contemplation (median = 6.4; IQR = 5.3, 7.9; *H* = 2.5, *df* = 1, *p* < 0.035). Action stage participants (median = 7.1; IQR = 6.5, 8.9), however, did not differ significantly from those in Preparation (*H* = 1.8, *df* = 1, *p* = 0.196) or Contemplation stages (*H* = 0.9, *df* = 1, *p* = 0.983)

### Body mass index and self-reported general health

**Fig. 2:**
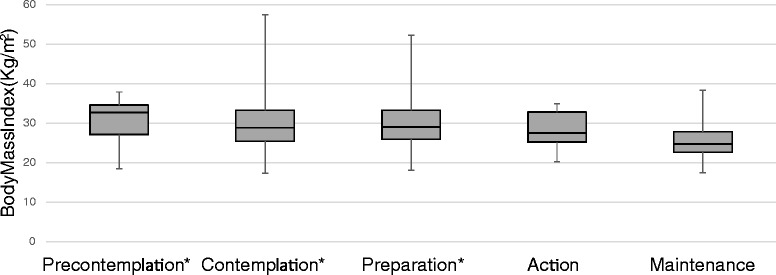
Body Mass Index by Stages of Change. As hypothesized, body mass index values differed by Stages of Change (*H* = 24.2, *df* = 4, *p* < 0.001). * Maintenance stage differed significantly from Precontemplation (*H* = 3.9, *df* = 1, *p* = 0.001), Contemplation (*H* = 5.6, *df* = 1, *p* < 0.001) and Preparation stages (*H* = 5.8; *df* = 1, *p* < 0.001

**Fig. 3:**
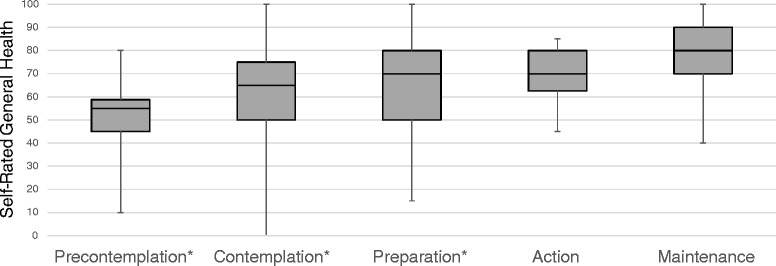
General Health by Stages of Change. As hypothesized, self-reported general health differed by Stages of Change (*H* = 31.2, *df* = 4, *p* < 0.001). * Maintenance stage differed significantly from Precontemplation (*H* = 5.4, *df* = 1, *p* = 0.001), Contemplation (*H* = 6.1, *df* = 1, *p* < 0.001) and Preparation stages (*H* = 5.4, *df* = 1, *p* < 0.001

Figure 2 displays median body mass index, and Fig. 3 shows self-reported general health values, across the five stages of change. Analyses showed that there was a significant difference across stages for body mass index (*H* = 24.2, *df* = 4, *p* < 0.001), as well as for general health (*H* = 31.2, *df* = 4, *p* < 0.001).

Consistent with hypotheses, those in Maintenance stage (median = 24.7 kg/m^2^, IQR = 22.6, 27.8) had lower body mass index values than participants in Precontemplation (median = 32.7 kg/m^2^, IQR = 27.1, 34.6; *H* = 3.9, *df* = 1, *p* = 0.001), Contemplation (median = 28.8 kg/m^2^, IQR = 25.4, 33.3; *H* = 5.6, *df* = 1, *p* < 0.001), and Preparation stages (median = 29.0 kg/m^2^, IQR = 25.9, 33.3; *H* = 5.8, *df* = 1, *p* < 0.001). Participants in the Maintenance stage were not significantly different from those in Action stage for body mass index or general health (*p* > 0.05).

As hypothesized, participants in the Maintenance stage (median = 80.0, IQR = 70.0, 90.0) reported better general health than their counterparts in Precontemplation (median = 55.0, IQR = 45.0, 58.8; *H* = 5.4, *df* = 1, *p* < 0.001), Contemplation (median = 65.0, IQR = 50.0, 75.0; *H* = 6.1, *df* = 1, *p* < 0.001), and Preparation stages (median = 70.0, IQR = 50.0, 80.0; *H* = 5.4, *df* = 1, *p* < 0.001).

## Discussion

This study used the baseline questionnaire, activity monitor, and anthropometric data from the Walk 2.0 Study to assess the criterion-related validity of a brief 10,000 steps per day Stages of Change (SoC-Step) instrument, and found substantial evidence for its validity. As hypothesized in accordance with the Transtheoretical Model, there were significant differences among the Stages of Change in steps per day, in likelihood of meeting the 10,000 steps per day goal, and in intention and self-efficacy related to achieving 10,000 steps per day.

According to Devon and colleagues [34], criterion-related validity is evidenced by the relationship between the characteristics of a measurement instrument and performance on another performance measurement. Concurrent criterion-related validity refers to the correspondence between scores from two instruments that were measured at the same point in time [34]. A measure achieves its degree of criterion validity (also known as empirical or statistical validity) to the extent that it corresponds with another observation that accurately measures the variable under study [35].

One key element of the SoC-Step instrument is the reported intention to “be physically active at a level of taking 10,000 steps or more on most days, if not all days of the week.” The Theory of Planned Behavior two-item scale of intention scores provided a concurrent measure of this construct. Consistent with theory, these intention scores varied as a function of the Stages of Change in the hypothesized direction, such that Precontemplation stage participants had lower levels of intention than those in Contemplation or Preparation. These findings support the concurrent validity of SoC-Step with regard to intention to meet the 10,000 steps per day standard.

In the present study, our criterion of physical activity was the daily step count obtained from ActiGraph activity monitors. The Stages of Change measure was significantly related to daily step count, as hypothesized. Most germane to the issue, those participants who indicated that, “Currently, I take enough steps (10,000 steps per day) to receive health benefits,” on the SoC-Step instrument (i.e., those classified in Action or Maintenance stages) had the highest step counts, as well as the highest likelihood of meeting the 10,000 steps per day standard. Together, these findings suggest that the SoC-Step instrument shows good concurrent validity with the Actigraph physical activity monitor criterion variable.

Self-efficacy is a central construct within both Social Cognitive Theory and the Transtheoretical Model. As hypothesized, the measures of physical activity self-efficacy pertaining to participants’ confidence to achieve varying levels of steps per day varied as a function of Stages of Change. The measure of self-efficacy pertaining to participants’ confidence to be active when faced with various challenges (self-efficacy to overcome common barriers to physical activity) was also related to Stages of Change in the hypothesized manner, although Action stage participants were not significantly different from those in Contemplation or Preparation (possibly due to the small number in Action stage). Overall, these findings provide further evidence for the criterion-related validity of the brief SoC-Step instrument.

The evidence for validity of the investigated SoC-Step instrument parallels that of other researchers who have previously developed and validated brief Stages of Change instruments [19, 20, 36–38]. The present instrument, however, is unique in its focus on the 10,000 steps goal. Reed and colleagues [37] investigated various algorithms to ascertain optimal characteristics of staging regular exercise behavior. These authors found that precise definitions of exercise, including all parameters needed to meet a criterion, were helpful to participants who were assessing themselves relative to a stage of change [37]. Furthermore, a five-choice format was endorsed as effective in assessing stage. The present study’s instrument conforms with these recommendations, in that extensive information with a precise behavioral definition is provided to the participant, along with a five-choice response format. The presence of such recommended characteristics may partially explain the substantial concordance observed between the SoC-Step and the criterion measure.

The observed relationships between stage classification and health are similar to systematic review findings by Bize et al. [39], along with those of Laforge et al. [40], who found that exercise stage was associated with self-perceived quality of life. In particular, Laforge et al. found that general health was lowest in Precontemplation, and highest in the Maintenance stage. Similarly, Cardinal and colleagues [36] found a relationship between Stages of Change in exercise and body mass index. The present study, however, is unique in its framing of Stages of Change around a public health physical activity goal, and in using additional objective health-related comparisons such as activity monitor data and clinically measured body mass index.

One limitation with regard to validity of this instrument was observed in that many participants who categorized themselves in Action or Maintenance stages for a physical activity level of 10,000 steps per day did not meet this standard based on objectively measured accelerometer data. It is important to note, however, that accelerometers do not capture all types of physical activity, and may provide a conservative estimate of step counts in some cases [31, 41]. In addition, our reliance on self-report instruments for both Stages of Change and intention may result in bias associated with common method variance, although undertaking measurement of participant intention without reliance on self-report would likely present alternate forms of bias.

Another limitation is the small number of participants classified in Precontemplation, which then hindered our ability to detect differences in relevant pairwise comparisons, due to low power. Having small numbers in Precontemplation is expected here, however, given that our study sample comprised those successfully recruited to a behavior change study. Although it may appear incongruous that participants recruited into a physical activity promotion study could classified in the Precontemplation stage, there are two issues to consider: 1) Stages of Change is specific to a target behavior [12], and while our behavioral target was 10,000 steps daily (at any intensity) for categorizing participants, they were originally included in the intervention if they “were currently engaging in less than a half an hour (30 min) of moderate-to-vigorous (e.g., walking, running or playing sport) physical activity on five or more days of the week;” 2) Stages of Change suggests that participants frequently regress into earlier stages [12], and that could have been a factor in the present study. Last, our sample was delimited to middle-aged Australians from two regions participating in an intervention (predominantly female, mostly overweight and obese, of a narrow age range, and mostly categorized in contemplation and preparation stages), so our findings may not apply to demographically different populations. Future studies should assess validity of the SoC-Step Instrument in younger adults.

Despite the aforementioned limitations, the present study possessed a number of strengths, including a large and diverse sample of middle-aged Australian adults who may represent a larger population of those who would be willing to participate in public health physical activity interventions. These participants provided not only self-report data, but also objective measures of physical activity and body mass index, which contribute to the rigor of this study’s methods.

## Conclusions

In this study, a cohort of mostly middle-aged, overweight and obese, Australian adults showed variations in objectively measured steps, body mass index, self-reported intention, and self-efficacy, as a function of self-reported Stages of Change toward the public health goal of achieving 10,000 steps daily. These variations corresponded with the hypotheses derived from the Transtheoretical Model, and thereby provided support for the criterion-related validity of the SoC-Step instrument. This brief instrument appears to have good criterion-related validity for determining Stages of Change related to the public health goal of 10,000 steps, and could be useful in tailored intervention efforts that could help lead to improvements in health-related quality of life.

## Additional file

Additional file 1:
**The SoC-Step, a Stages of Change instrument relevant to the physical activity goal of 10,000 steps per day.** (PDF 175 kb)

## Abbreviations

SoC-Step: The Stages of Change in Steps instrument related to 10,000 steps per day
SF-36: Short Form Health Survey
