# Supplementary material for: Validity of the Stages of Change in Steps instrument (SoC-Step) for achieving the physical activity goal of 10,000 steps per day
Source: BMC Public Health. 2015 Nov 30;15:1197. doi: 10.1186/s12889-015-2539-y (PMC4666193; doi:10.1186/s12889-015-2539-y)
Supplement: Additional file 1: — The SoC-Step, a Stages of Change instrument relevant to the physical activity goal of 10,000 steps per day. (PDF 175 kb) [file 12889_2015_2539_MOESM1_ESM.pdf]

INSTRUCTIONS: Before completing the questionnaire, please read the following information on the number of steps adults take each day.

1. **Both men and women can accumulate steps throughout the day in a number of ways:**
   - doing chores or working around the house
   - running errands or going to the shops
   - walking from place to place
   - doing exercise or playing sports
   - doing things such as going up the stairs, walking to the car, walking from one room to another, and so on.

2. **We might not know it, but large contributions to daily steps come from:**
   - having an active job (4000 to 9000 steps at work)
   - having an inactive or office job (1000 to 4000 steps at work)
   - a session of moderate-intensity walking or exercise (3000-4000 steps in 30 minutes of walking, which is more than 100 steps per minute)

3. **Studies have shown that:**
   - for healthy adults, jobs and the activities of daily living typically result in about 6000-7000 steps/day.
   - adults who do 30 minutes of walking or other exercise may attain an additional 3000-4000 steps/day.
   - most healthy adults accumulate between 3000 and 9000 steps per day.

4. **We can use the following categories to describe activity levels by steps:**

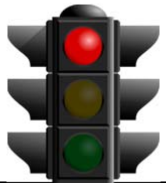

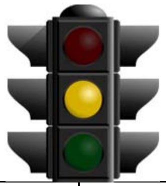

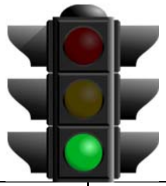

**Less than 5000 steps/day = <u>sedentary lifestyle</u>:**<br><br>typically minimal activities of daily living and inactive work, likely no sports/exercise	**5000-7499 steps/day = <u>low active</u>:**<br><br>typically some physical activity from activities of daily living on a work day, excluding sports/exercise	**7500-9999 steps/day = <u>somewhat active</u>:**<br><br>likely to have additional activity beyond activities of daily living from sports/exercise or active job	**10,000-12,499 steps/day = <u>active</u>:**<br><br>likely to have substantial additional physical activity beyond activities of daily living from sports/exercise or active job	**12,500+ steps/day = <u>highly active</u>:**<br><br>likely to have very substantial additional physical activity beyond activities of daily living from sports/exercise or very active job

Go on to the next page →

Stage of step taking

INSTRUCTONS: Please **tick only one box** below to indicate which the statements best applies to you:

☐	Currently, **I take enough steps** (10,000 steps per day) to receive health benefits.<br>AND<br>I have been physically active at this level **for more than SIX months**.
☐	Currently, **I take enough steps** (10,000 steps per day) to receive health benefits.<br>BUT<br>I have only been physically active at this level **for the past six months or less**.
☐	Currently, **I do NOT take enough steps** (10,000 steps per day) to receive health benefits.<br>BUT<br>**I do intend to** be physically active at a level of taking 10,000 steps or more on most days, if not all days of the week **within the next ONE month.**
☐	Currently, **I do NOT take enough steps** (10,000 steps per day) to receive health benefits.<br>BUT<br>**I do intend to** be physically active at a level of taking 10,000 steps or more on most days, if not all days of the week **within the next SIX months.**
☐	Currently, **I do NOT take enough steps** (10,000 steps per day) to receive health benefits.<br>AND<br>**I do NOT intend to** be physically active at a level of taking 10,000 steps or more on most days, if not all days of the week **within the next six months.**
